# Road Narrow‐Inspired Strain Concentration to Wide‐Range‐Tunable Gauge Factor of Ionic Hydrogel Strain Sensor

**DOI:** 10.1002/advs.202303338

**Published:** 2023-08-04

**Authors:** Wenyu Zhao, Zhuofan Lin, Zongtao Sun, Zhihao Zhu, Waner Lin, Yingtian Xu, Zhengchun Peng, Zhenglong Sun, Ziya Wang

**Affiliations:** ^1^ School of Science and Engineering The Chinese University of Hong Kong, Shenzhen Shenzhen 518172 China; ^2^ Center for Stretchable Electronics and Nano Sensors State Key Laboratory of Radio Frequency Heterogeneous Integration School of Physics and Optoelectronic Engineering Shenzhen University Shenzhen 518060 China; ^3^ Shenzhen Institute of Artificial Intelligence and Robotics for Society Shenzhen 518129 China; ^4^ Department of Micro‐Nano Electronics School of Electronic Information and Electrical Engineering Shanghai Jiao Tong University Shanghai 200240 China

**Keywords:** ionic hydrogel, strain concentration, tunable gauge factor

## Abstract

The application of stretchable strain sensors in human movement recognition, health monitoring, and soft robotics has attracted wide attention. Compared with traditional electronic conductors, stretchable ionic hydrogels are more attractive to organization‐like soft electronic devices yet suffer poor sensitivity due to limited ion conduction modulation caused by their intrinsic soft chain network. This paper proposes a strategy to modulate ion transport behavior by geometry‐induced strain concentration to adjust and improve the sensitivity of ionic hydrogel‐based strain sensors (IHSS). Inspired by the phenomenon of vehicles slowing down and changing lanes when the road narrows, the strain redistribution of ionic hydrogel is optimized by structural and mechanical parameters to produce a strain‐induced resistance boost. As a result, the gauge factor of the IHSS is continuously tunable from 1.31 to 9.21 in the strain range of 0–100%, which breaks through the theoretical limit of homogeneous strain‐distributed ionic hydrogels and ensures a linear electromechanical response simultaneously. Overall, this study offers a universal route to modulate the ion transport behavior of ionic hydrogels mechanically, resulting in a tunable sensitivity for IHSS to better serve different application scenarios, such as health monitoring and human–machine interface.

## Graphical abstract

A new geometrical configuration and mechanical combination strategy are proposed to improve the sensitivity of ionic hydrogel‐based strain sensors. The gauge factor of the PVA‐G‐FeCl_3_ ionic hydrogel‐based strain sensor can be adjusted between 1.31 and 9.21, and the electromechanical response remains linear simultaneously. This study offers a universal route to mechanically modulate the ion transport behavior of ionic hydrogels.

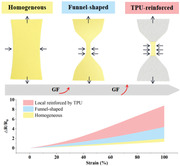

## 1 Introduction

Hydrogels, which consist of water‐rich polymer networks, can be used as ideal ionic conductors when mixed with electrolyte salts.^[^ ^1^ ^]^ Ionic hydrogels have received a lot of attention and have been used as bioelectronic bridges between electrons and biological systems because of their inherent capacity to stretch and be transparent as well as their superior processability and biocompatibility.^[^ ^2^ ^]^ However, the ionic hydrogel used as sensing elements like resistive strain sensors is still debatable compared to the “static” components, such as synthetic biological tissues, solid gel electrolytes, flexible bioelectronics backbones, and so on. One of the most significant obstacles holding ionic hydrogels back was their moderate electrical response, preventing strain‐induced deteriorated conductivity from obtaining high sensitivity like electronic conductors, and also precluding them from acting as strain‐insensitive resistance to maintain stable electrical transmission.^[^ ^3^ ^]^ To meet practical requirements, a universal approach for enhancing and adjusting the sensitivity of the ionic hydrogel‐based strain sensors (IHSS) is required (gauge factor [GF], as a critical figure of merit of sensitivity is defined as $\mathrm{GF}\;=\frac{\left(R-R_{0}\right)/R_{0}}{L-L_{0}}\;$, where *R* and *R*_0_ are the resistance before and after stretching, respectively, and *L* and *L*_0_ are the corresponding lengths before and after stretching, respectively).

To date, ionic conductivity has been adjusted to enhance the GF of ionic hydrogels. The conductivity of ionic hydrogels can be directly impacted by increasing ionic concentration and the value of the ion charge [Equation (S1), for further information see Note S1, Supporting Information].^[^ ^4^ ^]^ Generating ion modulation capability of the nanostructures and ion migration‐facilitated ion‐rich environment in the hydrogel network were also reported [Equation (S2), Supporting Information].^[^ ^5^ ^]^ However, the benefit of increasing the ion conductivity to increase GF is minimal compared to the electronic conductive strain sensors.^[^ ^6^ ^]^ This is because constructing channels for ion transport, altering ion species, or generating an ion‐rich environment can only reduce the initial resistance of the hydrogels. The conductivity maintains strain independence due to their inherent soft chain network, which cannot modulate ion transport behavior during elongation. As a result, their sensitivity is subject to *R/R*_0_ *= λ* ^2^ (*λ = L/L*_0_, see Note S2, Supporting Information, for details).^[^ ^7^ ^]^

Heterogeneous geometric configurations are considered to break the restrictions. Kim et al. extended the idea of microcrack structures to IHSS and developed a zigzag‐shape (macroscopic zigzags act as cracks) ionic sensor whose GF is vastly improved from 0.4 to 173 at 30% strain and maintains ≈50 at 200% strain.^[^ ^8^ ^]^ However, unlike the typical micro‐crack structure,^[^ ^9^ ^]^ where the resistance rises results from the interaction of strain‐generated countless cracks, the change of resistance of zigzag shape is caused by the separation of teeth from teeth. Therefore, the electromechanical response is nonlinear, resulting in a minimal reliable working range. In addition, Jiang et al. designed auxetic mechanical metamaterials and incorporated them into stretchable strain sensors to eliminate sensitivity limitations stemming from the Poisson effect.^[^ ^10^ ^]^ These metamaterials enhance 24 times the maximum sensitivity of a conductive single‐wall carbon nanotube‐based strain sensor. However, it only responds to a restricted range of strain (no more than 16%), and the electromechanical response is also nonlinear.

**Figure 1:**
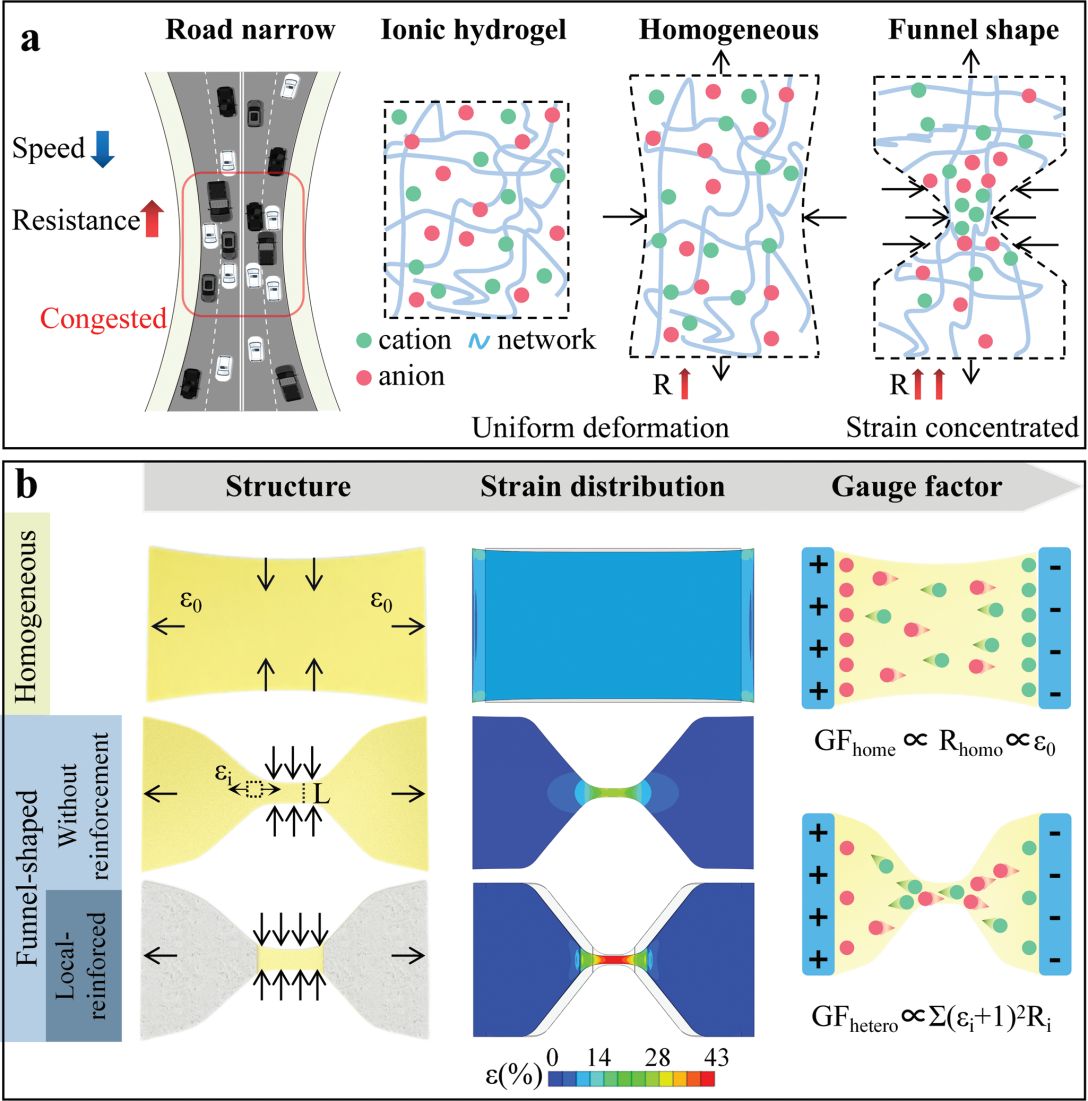
IHSS's tunable GF is based on strain concentration induced by funnel structure and TPU reinforcement. a) Road narrow‐inspired working mechanism of the geometry‐induced strain concentration for the IHSS's tunable GF. Ionic hydrogels contain crosslinked polymer networks and ion‐rich water. Stretching results in chain movement in the crosslinked network, which macroscopically appears as homogenous lateral contraction and longitudinal elongation, following Poisson's ratio. The “road narrow” effect is caused by the concentration of strain in heterogeneous systems where the geometric configuration varies. b) Schematic of a homogeneous structured ionic hydrogel under strain *ε*_0_; a funnel‐shaped ionic hydrogel, *L* represents the width of the narrow area and *ε_i_* is the local strain, and a TPU‐reinforced funnel‐shaped ionic hydrogel. Strain distribution analysis of homo‐, funnel‐shaped, and TPU‐reinforced funnel‐shaped structures was based on finite element modeling. Under strain *ε*_0_, the GF_homo_ of the homogeneous sensor depends on the resistance measured at both ends. In contrast, the GF_hetero_ of the funnel‐shaped sensor depends on the resistance measured at each section.

To solve the contradiction between sensor sensitivity and stretchability of the IHSS, strain concentration on hydrogel is considered to raise the volume change ratio during stretching gradually. **Figure** **1**a depicts the working mechanism of the geometry‐induced strain concentration affects ion transport modulation. Similar to how a narrowing road forces cars to slow down and change lanes, a more extreme geometric change in hydrogel under external forces causes a more substantial “road narrow” effect, resulting in a more noteworthy shift in ionic resistance and a higher sensitivity. More crucially, rather than surging, the modulating effect of the strain concentration on ion transport gradually increases with strain. As a result, the electromechanical response is linear, ensuring the working range of IHSS.

In addition, it is proved that the local strain *ε*_i_ can be improved when Young's modulus (*E*) reciprocal or section area (*A*) reciprocal in the resistance‐testing segment of the heterogeneous strain sensor is larger than the mean value^[^ ^11^ ^]^

(1) $$\varepsilon_{i}=\varepsilon_{i}\;\;\left(E_{i},A_{i}\right)=\frac{\varepsilon_{0}L}{E_{i}A_{i}\Sigma_{j=1}^{N}\frac{\Delta l}{E_{j}A_{j}}}\;$$

where *E_i_* and *A_i_* are Young's modulus and section area in segment *i*, and $\frac{\varepsilon_{0}L}{\Sigma_{j\;=\;1}^{N}\frac{\Delta l}{E_{j}A_{j}}}$ represents the force in segment *i*. Following this theory, we examined a universal strategy applicable to IHSS for wide‐range‐tunable GF by generating strain concentration from heterogeneous structures. Polyvinyl alcohol (PVA) is a hydrophilic, nontoxic, nonirritating polymer. PVA hydrogels can be prepared by a convenient freeze‐thaw crosslinking method, so it was chosen as the network for the ionic hydrogel.^[^ ^12^ ^]^ Glycerol (G), a well‐known nontoxic antidrying agent, was introduced as the cosolvent and ferric trichloride (FeCl_3_) served as the ion source. A funnel‐shaped structure corresponds to the control of *A* (as shown in Figure S1, Supporting Information), and a thermoplastic urethane (TPU) ‐reinforced strategy corresponds to the *E*, GF can be tunable by inducing and enhancing the strain concentration of PVA‐G‐FeCl_3_ hydrogel (Figure 1b). The potential GF‐affecting factors, including ion concentration, funnel structure parameters, and modulus after reinforcement, were investigated and discussed through simulations and tests as proof. Furthermore, a straightforward and general method for achieving high mechanical adhesion between hydrogels and elastomers was suggested. Overall, through the funnel structure and TPU reinforcement, the GF of this IHSS was significantly improved, and it can be continuously adjusted from 1.31 to 9.21 in the strain range of 0–100%.

## 2 Results and Discussion

**Figure 2:**
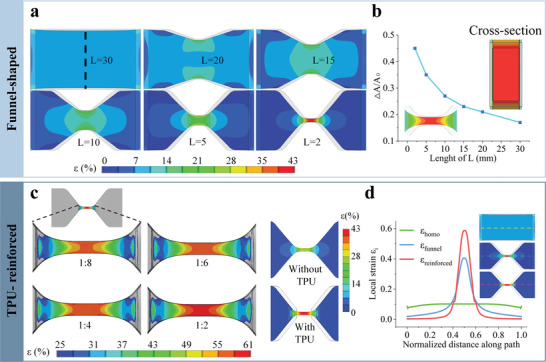
Ionic hydrogels with homogeneous and heterogeneous structures and their strain distribution. a) The strain distribution of funnel‐shaped hydrogels as determined by finite element analysis, with *L* = 30 mm, 20, 15, 10, 5, and 2 mm, *ε*_0_ = 10%. b) The variation in *L* of funnel‐shaped ionic hydrogels and the change in Δ*A/A*_0_, where *ε*_0_ = 10%. The insets show the frontal and sectional views of the narrow central section. c) Strain distribution of narrow central section of the TPU‐reinforced funnel‐shaped hydrogels with TPU:NaCl ratios of 1:2, 1:4, 1:6, and 1:8, *ε*_0_ = 10%. Hydrogels in the shape of funnel with or without TPU reinforcement are compared. d) The local strain distribution of homogeneous, funnel‐shaped, and TPU‐reinforced funnel‐shaped hydrogel films along the dotted line with *ε*_0_ = 10%.

Equation (1) shows that *ε_i_* is related to both *E* and *A*. Thus, various geometric designs have been attempted to achieve strain concentration by modifying *A* first (Figure S2, Supporting Information). A funnel‐shaped structure performs the best due to its largest shift in local geometry. **Figure** **2**a depicts the strain distribution of the homogeneous and funnel‐shaped ionic hydrogels (*L* = 2, 5, 10, 20, 30 (mm), *ε*_0_ = 10%). When the homogeneous ionic hydrogel is stretched, the local strain disperses uniformly and keeps constant for each differential length of the film (*ε_i_* = *ε*_0_). The resistance of each differential segment is: *R_i_ = λ^2^R*_0_ *_i_*, therefore, *R*_homo_ *=* Σ(ε_*i*_ + 1)^2^ *R*_0*i*_ = (ε_0_ + 1)^2^ *R*_0_, according to the relation *R/R*_0_ *=* λ^2^. Thus, the GF_homo_ has a positive correlation with *ε*_0_. In comparison, when the funnel‐shaped hydrogel was stretched to 10% strain, the narrow central section experienced concentrated strain. In contrast, the local strain on both sides is less than 10%. The *ε*_max_ changed from 10% to 14% as *L* decreased from 30 to 10 mm, and drastically increased from 14% to 41% as *L* decreased from 10 to 2 mm. Furthermore, the change of Δ*A*/*A*_0_ with *L* in Figure 2b shows a similar trend, implying a more significant strain concentration with the alteration of local geometry, i.e., a more significant “road narrow” effect and a greater GF. In addition to increasing local elongation, strain concentration also raises local resistance with the sectional area [Equation (S3), Supporting Information].

*E* can also affect how local strains are distributed, in addition to *A*. In order to strengthen the hydrogel, a porous TPU was created and mechanically attached to both sides of the hydrogel (Figure S3, Supporting Information). More importantly, its modulus may be widely adjusted by varying the NaCl content (the mass ratio of TPU to NaCl during manufacture is 1:2, 1:4, 1:6, or 1:8), fulfilling the requirement to adjust the GF of hydrogels. According to the depiction in Figure 2c of the related strain distribution, TPU reinforcement can further enhance strain concentration based on the funnel‐shaped hydrogel. In comparison, TPU inhibits the strain from propagating to both sides and concentrates it in the center instead (Movie S1, Supporting Information). The highest local strain found in the narrow center portion increases from 50% to 61% when the NaCl content drops from 1:8 to 1:2 (the modulus values used for the finite element simulation are acquired experimentally and illustrated in the next section). The local strain distribution for homogeneous and funnel‐shaped with/without TPU sheets is shown in Figure 2d along the dotted line. From the finite element simulation results, *ε_i_* = *ε*_0_ everywhere for the homogeneous film. As for the two heterogeneous films, we have *ε_i_* >> *ε_0_* at the strain concentration section, and *ε_i_* < *ε_0_* on both sides of the film. This trend intensified when TPU was introduced, indicating a more significant “road narrow” effect.

**Figure 3:**
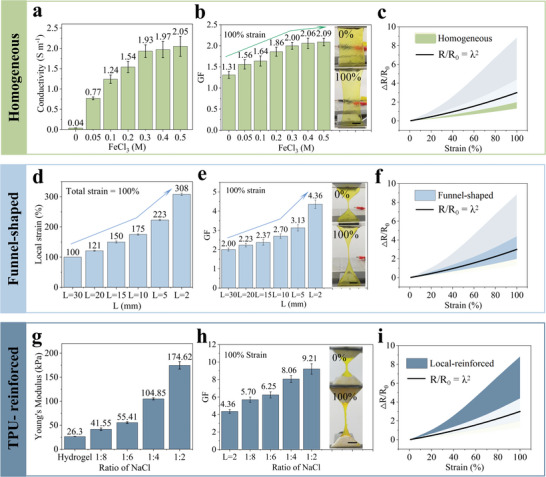
Tests on homogeneous and heterogeneous PVA10‐G30‐FeCl_3_ hydrogels’ related properties and GF range. a) Homogeneous hydrogel conductivity at various ion concentrations. b,c) GF of homogeneous hydrogels at 100% strain with different ion concentrations. The inset shows the diagrams of the stretched hydrogels, scale bar: 1 cm. GF cannot break the theoretical limit by increasing ion concentration. d) The local strain of the narrow central section of funnel‐shaped PVA10‐G30‐FeCl_3_ hydrogels, as the total strain is 100%. e,f) GF of funnel‐shaped PVA10‐G30‐FeCl_3_ hydrogels at 100% strain. Scale bar: 1 cm. The funnel structure‐induced strain concentration generated a more significant “road narrow” effect, breaking through the theoretical value of GF. g) Young's modulus of the TPU‐reinforced PVA10‐G30‐FeCl_3_ hydrogels. h,i) GF of the TPU‐reinforced funnel‐shaped PVA10‐G30‐FeCl_3_ hydrogels at 100% strain. Scale bar: 1 cm. The local reinforcement of TPU makes the strain more concentrated, and GF further increases on the basis of the original funnel structure.

The content of each component influences the PVA‐G‐FeCl_3_ hydrogel's mechanical and electrical properties (see details in Note S3, Supporting Information). After comprehensively considering its conductivity and stretchability (Figures S4–S6, Supporting Information), PVA10‐G30 (the number represents the mass percentage of the substance, same as follows) is selected. The GF of PVA‐G‐FeCl_3_ hydrogels with different components, geometric structures, and different levels of reinforcement were examined. **Figure** **3**a,b and Figure S7a (Supporting Information) demonstrate the effects of ion concentration on the conductivity and GF of hydrogels with a homogeneous structure under 100% strain. By introducing ions (Fe^3+^ and Cl^−^), the conductivity of PVA10‐G30 hydrogel was significantly enhanced. Conductivity rapidly increases from 0.04 to 1.93 S m^−1^ (*f* = 50 Hz) when ion content climbs from 0 to 0.3 m. An increase in ion concentration means that there are more ions in the network per unit volume, which leads to a rise in the number of charges per unit time per unit cross‐sectional area (*n*) when an electric field is applied [Equation (S1), Supporting Information]. However, the few water‐containing networks can support a finite amount of freely mobile ions. A plateau is reached, as shown in Figure 3a, when the ion concentration reaches a critical level (in this example, over 0.3 m). At this point, *n* no longer varies when the electrolyte concentration increases.^[^ ^13^ ^]^ Similarly, the GF of PVA10‐G30 hydrogel grew as the ion concentration increased until it reached a plateau at the ion concentration of 0.3 m (GF = 2.00, Figure 3b and Figure S7a, Supporting Information). This is probably because the conductivity variation determines its initial resistance *R*_0_, affecting its GF according to $\mathrm{GF}\;=\frac{\left(R-R_{0}\right)/R_{0}}{\varepsilon }\;$. However, the deformation results from the change in the configuration of the PVA network, negligibly affecting ion transport pathways. In other words, the geometric change *L/S* is primarily responsible for the difference in the resistance of the stretched ionic hydrogel. Consequently, as illustrated in Figure 3c, the GF of the homogeneous ionic hydrogel is confined to a limited range and follows *R/R_0_ =* λ*^2^*.

Secondly, hydrogels were measured by the GF of funnel‐shaped PVA‐G‐FeCl_3_ (the concentration of FeCl_3_ is 0.3 m, same as follows). Figure 3d depicts the local strain of the narrow central section as the funnel‐shaped films were stretched to 100% total strain. As *L* decreases, the local strain increases linearly (from *L* = 30 mm to *L* = 10 mm), verifying the effectiveness of generating strain concentration through geometric configuration. And then, it abruptly achieves the maximum (from *L* = 10 mm to *L* = 2 mm) because of a more significant strain concentration. Besides, as depicted in Figure 3e, Figures S7b and S8 (Supporting Information), the trend of GF increasing with *L* reducing is more significant (from 2.00, *L* = 30 mm to 4.36, *L* = 2 mm), showing the inherent logical relationship between local strain and GF and confirming the validity of the “road narrow” effect. In addition, all the ionic hydrogels’ resistance increases linearly with tensile strain over the entire strain range (0–100%), indicating that the “road narrow” effect occurs gradually rather than suddenly during stretching.

In addition, by increasing the local modulus via the adhesive elastomer, the local strain and GF of the funnel‐shaped hydrogel can be further enhanced. Figure 3g exhibits Young's modulus of bare PVA10‐G30‐FeCl_3_ hydrogel and porous TPU‐adhesived hydrogels with different NaCl proportions. The hybrid's Young's modulus can be easily varied by decreasing the NaCl content and can be highly enhanced up to 174.62 kPa (TPU:NaCl = 1:2). The GF of funnel‐shaped PVA10‐G30‐FeCl_3_ with varying degrees of reinforcement were tested and the results were shown in Figure 3h and Figure S7c (Supporting Information). TPU reinforcement considerably increased GF at 100% strain (TPU:NaCl = 1:8, GF = 5.70) when compared to bare hydrogel (*L* = 2 mm, GF = 4.36), and it continued to expand until the hybrid modulus reached its maximum (TPU: NaCl = 1:2), at which point GF also reached its maximum of 9.21. Consistent with the simulation results, local reinforcement by porous TPU can further promote the strain concentration of funnel‐shaped hydrogels, thereby increasing GF and beyond the theoretical limit through the “road narrow” effect, as shown in Figure 3i. More importantly, the funnel structure and TPU reinforcement allow for constant changes in strain concentration. As a result, it is possible to steplessly modify the GF of PVA10‐G30‐FeCl_3_ hydrogels between 1.31 and 9.21 while preserving excellent linear response.

**Figure 4:**
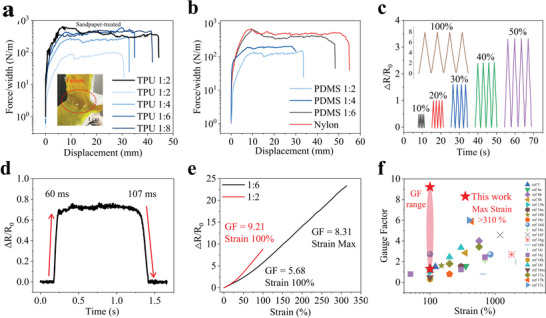
Hydrogel–elastomer interface tensile test and sensing capability of the TPU‐reinforced funnel‐shaped IHSS. a) The hydrogel–TPU interface's observed peeling forces per width. Sandpaper was utilized to increase the elastomers’ surface roughness. The proportion displays the mass proportion of TPU to NaCl. The permeability of the hydrogel solution, the porosity of the porous TPU, and the interface bonding are all increased with an increase in the dosage of NaCl. The inset shows that the hydrogel bulk undergoes a cohesive failure during the peeling test, leaving a thin residual layer of hydrogel on the TPU substrates. b) The hydrogel–elastomer interface's observed peeling forces per width. Similar techniques were used to create the porous PDMS, which was combined with hydrogel to generate a mechanical interlock. c) Response and recovery time of the TPU‐reinforced funnel‐shaped IHSS. d) Cyclic stability tests of the TPU‐reinforced funnel‐shaped IHSS under strain from 10% to 100%. e) The maximum GF at maximum strain and the maximum GF at 100% strain as produced by TPU‐reinforced (1:2) funnel‐shaped IHSS and TPU‐reinforced (1:6) funnel‐shaped IHSS, respectively. f) A comparison between the GF in this study and previously reported IHSS.

Due to their different moduli, the hydrogel–elastomer interface will experience lateral shear stress as the strain sensor deforms. Because PVA hydrogel frequently has inadequate adhesion energy with commonly used elastomers (≈1 J m^−2^), the stress generated at the interface is intolerable, especially when subjected to severe strains. This causes interface separation, which will lower the accuracy and service life of the sensor. To address this issue, we propose a straightforward yet all‐encompassing method to produce porous TPU (see details in the Experimental Section) and assemble hydrogels into hierarchical structures with incredibly robust interfaces. Specifically, hydrogel solution can soak into the micropores on the surface of the TPU layer, and after crosslinking, the hydrogel was mechanically interlocked on the surface of the porous TPU layer (Figure S9a, Supporting Information).^[^ ^14^ ^]^ The standard 90°‐peeling test was conducted to quantitatively assess the robustness of the hydrogel–TPU hybrid, as illustrated in **Figure** **4**a and Figure S9b (Supporting Information). The effect of different NaCl mixing ratios of TPU on the interfacial toughness was tested, and the steady‐state peeling force per unit width of the hydrogel was used to characterize the interfacial toughness of the hybrid. On the one hand, the NaCl content directly influences the density and size of pores in porous TPUs, as shown in Figure S10 (Supporting Information). Therefore, a higher NaCl ratio is more favorable for porous TPU infiltrating the hydrogel solution and forming a stronger interface. As a result, when the NaCl ratio is insufficient, interfacial separation occurs (TPU:NaCl = 1:2 or 1:4, the interfacial toughness is 80 and 260 J m^−2^, respectively). Once the ratio exceeds 1:6, hydrogel destruction takes the place of peeling, and the peeling curves reach their maximum level of >400 J m^−2^. This represents the fracture energy of this PVA‐G‐FeCl_3_ hydrogel. On the other hand, a high NaCl level reduces the sensor's adjustable GF range and weakens the strain concentration caused by TPU strengthening. It is believed that increasing the roughness and the contact area between the hydrogel and TPU can achieve a stronger interlocking interface, therefore, sandpaper was utilized as the template to fabricate rough porous TPU (TPU:NaCl = 1:2).^[^ ^14^, ^15^ ^]^ A higher interfacial strength was successfully obtained, as demonstrated in Figure 4a, the crack propagated at the hydrogel bulk rather than at the hydrogel‐TPU interface, leaving a residual layer of hydrogel on the TPU substrate. This indicates that the toughness of the interface exceeds that of the hydrogel itself. Besides, other porous elastomers such as polydimethylsiloxane (PDMS) manufactured by the same method, and commercially available nylon fabric can also form interlocking interface with the hydrogel. As shown in Figure 4b, the interfacial toughness of hydrogel‐PDMS hybrid increases with the proportion of mixed NaCl particles and exceeds the fracture energy of the hydrogel at 1:6. The effect of adhesive porous PDMS and nylon fabric on the mechanical and sensing properties of hydrogels is similar to that of TPU (Figure S11, Supporting Information). While TPU is still the ideal reinforcement material for the sensor due to its improved stretchability, larger modulus adjustable range, and stronger interfacial toughness.

Figure 4c–e shows the sensing qualities of the TPU‐reinforced sensor and demonstrates the feasibility of the TPU‐reinforced strategy from the performance perspective. First of all, while stretching the sensor, a small amount of deformation might result in a change in resistance, and as shown in Figure 4c, the reaction time and recovery time were found to be 60 and 107 ms, respectively. Due to its fast response time, high robustness and stability, the sensor shows repeatable curves under different cyclical strains in the 10%–100% range, as shown in Figure 4d. The GF of the sensor will increase with the increase of strain concentration, but the elongation at break will decrease. Although a sensor with *L* = 2 mm, TPU:NaCl = 1:2 can withstand 100% strains, sensitivity has to somewhat be sacrificed if a larger operating range is required. As seen in Figure 4e, when the ratio of TPU to NaCl is less than or equal to 1:6, a maximum strain beyond 310% is reached at the expense of a fall in the sensor's GF from the highest value of 9.21 (100% strain) to 5.68 (100% strain). While both sensors maintain a linear electromechanical response until break, allowing for the detection and quantitative differentiation of each strain range. In terms of two critical parameters for stretchable strain sensors, GF and available strain limit, the performance of the TPU‐reinforced funnel‐shaped ionic hydrogels was compared to those of previously reported IHSS (Figure 4f and Table S1, Supporting Information). The GF of the majority of strain sensors was considerably worse.^[^ ^3^, ^4^, ^5^, ^13^, ^16^ ^]^ Other strain sensors with high GF were only produced at maximum strain (far more than 100%), and they lacked a linear response.^[^ ^4^, ^17^ ^]^

**Figure 5:**
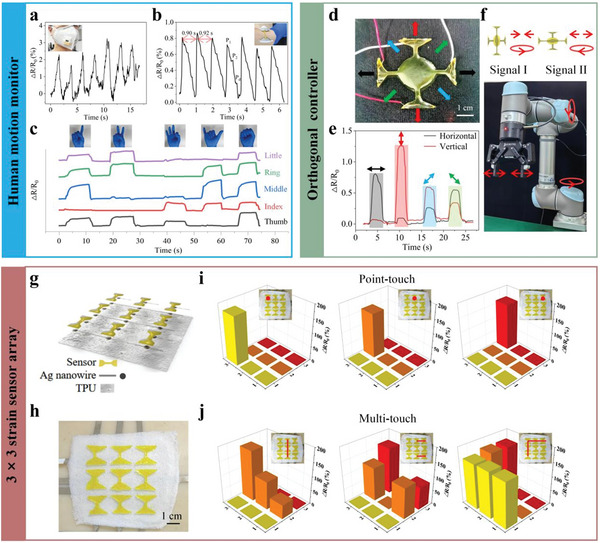
Applications of the TPU‐reinforced funnel‐shaped IHSS. a) Breath detection by fixing the sensor on a mask. b) Real‐time radial artery pulse monitoring by attaching the sensor to the wrist. Each radial artery pulse waveform contains “P_1_,” “P_2_,” and “P_3_” peaks. c) Gesture recognition for communication. d) Photograph of the orthogonally arranged sensors. Direction‐dependent signals can be detected when it was stretched in different directions. e) The anisotropic response of the orthogonally arranged sensors. The magnitude of the difference between two rates of resistance change (*R*_h_ − *R*_v_) can be used to determine the direction of the force. f) Photograph of the two‐finger claw and the robot arm. The claw can realize the opening and closing motion, and each degree of freedom of the robot arm can rotate forward or backward according to the positive and negative signals of *R*_h_ − *R*_v_ produced by different stretching directions. g) Schematic illustration of the structure of the 3 × 3 strain sensor array. Ag nanowire was coated on the porous TPU layer as the electrodes. h) Photograph of the 3 × 3 strain sensor array. i) Point‐touch functionality of the array exhibiting a positive change in resistance as each sensor was touched. j) Detection of three‐, four‐, and five‐finger touch simultaneously, demonstrating multitouch capability.

The TPU‐reinforced funnel‐shaped IHSS was developed for various applications due to its excellent mechanical properties, high sensitivity, orientation‐dependent response, and signal stability in the 0–100% strain range. First of all, the sensor's high sensitivity and quick response capability enable it to detect subtle human motions with great detail. As shown in **Figure** **5**a, the changes in the ∆*R*/*R*_0_ signals can be recorded in real time when the sensor is attached to a mask to monitor the airflow produced by breathing. In addition, it is possible to find the human pulse (Figure 5b). Three wave components, the percussion wave, the tidal wave, and the diastolic wave (P_1_, P_2_, and P_3_), are readily seen in a typical radial pressure waveform. These physiological indexes are crucial for clinical diagnosis in the present‐day medical field. Moreover, we look into how the suggested sensors might be used to help those with disabilities. Figure 5c shows how gesture recognition was made possible by combining five sensors with a nitrile glove. This method can help deaf and mute people as well as those without a sign language background communicate more effectively.^[^ ^18^ ^]^ In addition, a four‐finger hand strain‐sensing device that can identify grips was demonstrated (Figure S12, Supporting Information). Each bottom knuckle of the fingers was fitted with a TPU‐reinforced funnel‐shaped IHSS, which acquired the characteristic signals associated with picking up several fruits (coconut, apple, banana, orange, and peach).

In addition to porous TPU, nylon fabric can also act as a reinforcer, which has a strong interface with the hydrogel, and exhibit improved mechanical and sensing properties (Figure S11b,c, Supporting Information). This hydrogel‐nylon fabric hybrid can be used for the flexible human–machine interface, serving as an input device for control signal of multi‐axis (multi‐degree of freedom) robotic manipulators and mechanical claws to complete grasping tasks (Figure 5d–f and Movie S2, Supporting Information). We used a stretchable nylon fabric as the sensor substrate to verify the generality of making mechanical interlocking between the hydrogel and porous elastomers. We merged two sensors with an orthogonal arrangement to sense the direction of strains based on the anisotropic resistive response characteristic of the funnel‐shaped sensor (Figure 5d). As illustrated in Figure 5e, the strain applied at 0° (parallel to the sensor in the horizontal arrangement) resulted in the positive value of the difference between two rates of resistance change *R*_h_ – *R*_v_; in contrast, a strain at 90° causes a negative value of *R*_h_ − *R*_v_. These two signals, produced by stretching in various directions, can be used to control the mechanical claw's opening and closing and the arm's forward and backward motion in multiple degrees of freedom (Figure 5f). A custom MATLAB program is used to access the control permissions of different items through a converter button.

To further demonstrate the potentials of the TPU‐reinforced funnel‐shaped IHSS for external stimuli detection, a 3 × 3 IHSS array based on porous TPU was developed for sensing external stimuli distribution (Figure 5g–j and Movie S3, Supporting Information). Isopropyl alcohol‐dispersed silver nanowires were used to draw the circuit onto the porous TPU layer. The resultant ∆*R*/*R*_0_ reflects the location of the stimuli when a single finger touch was applied to the top stacked sensors of the array (inset of Figure 5i). Figure 5j shows how the array can recognize three, four, and five fingers, demonstrating its multitouch capability. Furthermore, the funnel structure increases the sensitivity of each sensor in the middle, resulting in negligible ∆*R*/*R*_0_ signal from neighbor sensors in the maps, enabling reconstruction of the strain distribution with higher resolution.

## 3 Conclusion

Inspired by the narrowing of the road, a universal strategy to modulate ion transport through strain concentration was proposed to achieve stepless tunable GF of IHSS. The adoption of a funnel structure with TPU reinforcement produced controllable strain concentration. As a result, the PVA‐G‐FeCl_3_ IHSS with GF from 1.31 to 9.21 (under 100% strain) can be obtained and the electromechanical response remains linear simultaneously. In addition, we proposed a method to fabricate moduli controllable porous elastomers, such as TPU, PDMS, and Nylon, which realize solid mechanical adhesion with the ionic hydrogel. The interfacial toughness of TPU and PVA‐G‐FeCl_3_ is more than 400 J m^−2^, which is higher than the fracture energy of hydrogel itself, ensuring the mechanical robustness, high elasticity and sensitivity of the sensors. Consequently, the TPU‐reinforced funnel‐shaped IHSS reaches a maximum GF of 9.21, a maximum strain of 310%, and high conductivity of 1.93 S m^−1^. Human motions, such as breathing and pulse were monitored. Relying on the anisotropic resistive response characteristic of the IHSS, we invented an orthogonal controller and applied it to control the robot arm and claw to grasp objects. In addition, an IHSS‐integrated array to obtain strain maps was also demonstrated. The results show that the combination of structural engineering and device design of ionic hydrogel can achieve strain concentration and ion transport modulation to breakthrough theoretical upper limit of GF of IHSS, which has significant application value for ionic hydrogels in health monitoring and human–machine interface.

## 4 Experimental Section

### Materials

PVA (average Mw 130 000, 99+% hydrolyzed; Sigma‐Aldrich), glycerol (ACS, ≥99.5%; Aladdin), FeCl_3_·6H_2_O (Aladdin), NaCl (AR, 99.5%; Aladdin), TPU (commercially available), nylon fabric (commercially available), PDMS elastomer (Sylgard184; Dow corning), and isopropyl alcohol dispersed silver nanowires (Suzhou ColdStones Tech Co., Ltd.). Unless otherwise specified, the water used in this experiment is deionized water (MΩ at 25 °C).

### Fabrication of PVA, PVA‐G, and PVA‐G‐FeCl_3_ Ionic Hydrogels

All the PVA‐based hydrogels were prepared by one time of freezing and thawing. Different content of PVA solutions (8, 10, 12, 15, and 18 wt%) were prepared by dissolving PVA powder in deionized water under heating (100 °C) and magnetic stirring for 2 h. Then the PVA solutions were poured into the mold and cooled at −20 °C for 12 h. For exploring the influence of glycerol content on Young's modulus of the PVA‐based hydrogel, a series of PVA‐G solutions (PVA10‐G10, PVA10‐G20, PVA10‐G30, and PVA10‐G40, the number represent the percentage of the mass of the components) were prepared by dissolving PVA powder in a mixture of deionized water and glycerol. After heating and stirring, the PVA‐G solutions were poured into the mold and cooled at −20 °C for 12 h. PVA‐G‐FeCl_3_ hydrogels were prepared by mixing FeCl_3_ particles into the PVA10‐G30 solutions and magnetic stirring for 2 h. Then the PVA‐G‐FeCl_3_ solutions were poured into the mold and cooled at −20 °C for 12 h as well.

### Fabrication of the TPU‐NaCl Porous Elastomer

Commercially available TPU particles were dissolved in a N,N‐dimethylformamide (DMF) solvent first. Then NaCl particles with a weight ratio of 1:2, 1:4, 1:6, and 1:8 (TPU:NaCl) were added as the sacrificial template and stirred using a planetary vacuum mixer (HM800, HASAI). Then these slurries were fully filled into molds with 60 Cw sandpaper at the bottom and cured at 80 °C for 4 h in an oven. After that, the cross‐linked TPU‐NaCl mixture were released from the mold and soaked into deionized water (fill a 1 L beaker) for 12 h to completely the NaCl particles; the water was refreshed every 2 h.

### Fabrication of the Funnel‐Shaped TPU Reinforced Hydrogels

A funnel structure was designed, as shown in Figure S1 (Supporting Information), and the funnel‐shaped molds were fabricated by 3D printing (Sermoon D1, Creality). A porous TPU layer was cut into the funnel shape and placed on the bottom of the mold, then the PVA10‐G30‐FeCl_3_ solution was poured into the mold, vacuumed for 5 min to immerse the solution into the porous TPU layers, and then frozen at −20 °C for 12 h to cross‐link.

### Characterization

Fourier transform infrared (FTIR) spectroscopy was performed using an FTIR spectrometer Nicolet 560. The X‐ray diffractometer (XRD) tests of the PVA‐G hydrogels were carried out on the Rigaku SmartLab at 2θ from 5° to 40° using Cu Kα radiation (*λ* = 0.154056 nm) at a scanning speed of 5° min^−1^. The morphologies and structures of the PVA‐G hydrogels were characterized by an Apreo 2S scanning electron microscopy (SEM). All hydrogel samples were freeze‐dried in a vacuum (Scientz, 12N) for over 48 h. Then samples were cut to expose the cross‐section and sputtered with gold for observation. The interlocking structure of hydrogel–TPU hybrid, the porous structure of TPU, and the 3D image of porous TPU with/without sandpaper were observed by an Optical Microscope Leica DM6 B.

### Mechanical Tests

The mechanical property of hydrogels with different PVA, G and ion contents, and TPU‐adhesived hydrogels, and the interface toughness of hydrogel–elastomer hybrid were tested using an INSTRON 5943 universal testing machine. All stretched samples' length, width, and thickness were 50 mm × 30 mm × 1.2 mm, and the stretching rate was 0.5 mm s^−1^. The Young's modulus (*E*) was calculated by dividing the applied stress (*σ*) by applied strain (*ε*)

(2) $$E\;=\frac{\sigma }{\varepsilon }\;=\frac{F/S}{\Delta L/L_{0}}\;$$

where *F* is the tensile force, *A* is the cross‐sectional area, Δ*L* is the length variation, and *L*_0_ is the initial length of the sample. The hydrogel used in all the 90°‐peeling tests is PVA10‐G30‐0.3 M. The upper elastomer was glued with a thin polyethylene (PE) film as a stiff backing to prevent elastic stress during testing. The bottom layer was glued to a ball bearing to ensure the peeling angle during the test.

### Electrical Tests

A digital multimeter (Keithley DMM6500) was used to measure the electrical signal of the hydrogels. When the hydrogel sample was stretched, two electrodes were attached at both ends and connected to the multimeter. The resistance change with time was obtained as the sample was tested with a speed of 0.5 mm s^−1^. The strain sensitivity of hydrogel‐based sensors was calculated by GF, as defined by the ratio of the relative resistance change rate to the applied strain $\mathrm{GF}\;=\frac{\left(R-R_{0}\right)/R_{0}}{\varepsilon }\;$.

The ionic conductivity of each hydrogel film was determined via an AC perturbation method by clamping between two stainless steel plate electrodes. The ionic impedance was determined using an LCR meter (Keysight, E4980AL) by applying 10 mV AC at 50 Hz. All measurements were carried out at room temperature. The conductivity, *σ*, was then determined from the impedance using the equation: *σ = t/RA*, where *t* is the thickness of the hydrogel (separation between the metal plates), *R* is the real part of the impedance, and *A* is the area of the film filled by the sample.

## Conflict of Interest

The authors declare no conflict of interest.

## Supporting information

Supporting Information

Supplemental Movie 1

Supplemental Movie 2

Supplemental Movie 3

## Data Availability

The data that support the findings of this study are available from the corresponding author upon reasonable request.
