# Supplementary material for: Road Narrow‐Inspired Strain Concentration to Wide‐Range‐Tunable Gauge Factor of Ionic Hydrogel Strain Sensor
Source: Adv Sci (Weinh). 2023 Aug 4;10(28):2303338. doi: 10.1002/advs.202303338 (PMC10558700; doi:10.1002/advs.202303338)
Supplement: Supplementary file 1 — Supporting Information [file ADVS-10-2303338-s003.pdf]

ADVANCED SCIENCE
Open Access

Supporting Information

for *Adv. Sci.*, DOI 10.1002/advs.202303338

Road Narrow-Inspired Strain Concentration to Wide-Range-Tunable Gauge Factor of Ionic Hydrogel Strain Sensor

Wenyu Zhao, Zhuofan Lin, Zongtao Sun, Zhihao Zhu, Waner Lin, Yingtian Xu, Zhengchun Peng, Zhenglong Sun and Ziya Wang**

Supporting Information

Road Narrow-inspired Strain Concentration to Wide-range-tunable Gauge Factor of Ionic Hydrogel Strain Sensor

Wenyu Zhao, Zhuofan Lin, Zongtao Sun, Zhihao Zhu, Waner Lin, Yingtian Xu, Zhengchun Peng, Zhenglong Sun, and Ziya Wang**

Note S1. **Ionic conductivity**

For ionic conductors, carriers are negatively charged anions or positively charged cations. There can be many kinds of ions with different charges, so the total

conductivity of an ionic conductor is given by the sum of all the carrier contributions:[1]

$$\rho=\Sigma_i Z_i e n_i u_i \quad (1)$$

Where Z_i is the absolute value of the ion charge, e is the fundamental charge, n_i is the charge carrier density, and μ_i is the mobility for each ion. It shows that increasing the ion concentration can only increase the initial conductivity, rather than $\Delta\rho$ under tension.

In addition, in hydrogel networks, the diffusion coefficient of ions is smaller than that in water, which needs to be adjusted according to pore size and topography:[2]

$$D_{\mathrm{eff}} = \frac{D_0 \varepsilon}{\tau} \quad (2)$$

Where D_{eff} is the effective diffusion coefficient in a porous network, D_0 is the diffusion coefficient in liquid, ε is the porosity, and τ is the tortuosity. Therefore, the mobility of ionic species is greatly affected by the network topology of nanoscale ionic conductors. In nanofluids, τ describes the non-linear path from one side of the membrane to the other.[3] Highly ordered or longitudinally aligned ion-insulated nanostructures can provide low-bending paths to facilitate ion transport, resulting in significantly lower τ, and thus improved conductivity.[4]

Note S2. Limitation of ionic hydrogel's GF

External stimulus can change the cross-linking network configuration or pore shrinkage of the ionic hydrogel. However, these changes do not/hardly affect the

modulation effect of ion transportation or conductive pathway as significantly as in the case of electronic conductive materials. Therefore, the ionic conductivity does not change or only slightly increases as stretched due to the preferential orientation of elastic chains.[5] The electrochemistry of hydrogels is similar to that of aqueous electrolytes,[6] and the response of ionic hydrogels to strain can be quantified by a piezoresistor which follows the equation:

$$R = \frac{\rho L}{A} \tag{3}$$

Where R is the bulk resistance, ρ is the conductivity, L and A are the length and cross-sectional area of the hydrogel. Therefore, the change in the resistance of the ionic gel during the stretching process mainly comes from the shape change L/A rather than the change in the conductivity. Thus, the GF of the ionic gel is confined to a small range and follows $R/R_0 = L/L_0 = \lambda^2$.[7]

Note S3. Characterization and analysis

The content of PVA can directly affect the mechanical and electrical properties of hydrogels, as shown in Figure S3a and b. PVA10 (the number represents the mass percentage of the substance, same as follows) was decided to be used in this study due to its greatest elongation at break and sufficient water content. Glycerol can significantly improve the mechanical strength, extensibility, toughness, and stiffness of the PVA hydrogel and slightly affect its GF (Figure S3c and d). Because the glycerol-water binary system introduced more noncovalent interactions (such as hydrogen bonds, (Figure S4a) in the networks, which can act as sacrificial bonds to dissipate external energy during deformation efficiently.[8] In addition, the crystallization of PVA is promoted in the co-solvent environment and the crystallinity of PVA-G hydrogels is much higher than that of PVA hydrogel (Figure S4b). As physical cross-linking points, the PVA crystals can be rearranged or even ruptured to further dissipate energy at a high strain. Moreover, with the increase of glycerol, interconnected tiny pores can be formed and multiplied in the hierarchical porous architecture of PVA hydrogels (Figure S5, Supporting Information). This hierarchical porous architecture acts as a well-connected network of percolation paths, facilitating the diffusion and migration of ions.[8] However, the amount of glycerol is restricted to ensure adequate water content to support the movement of a large amount of ions. Therefore PVA10-G30 was selected as the most comprehensive option.

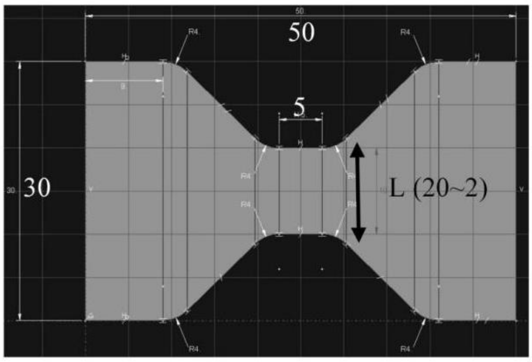

Figure S1. The geometrical parameter of the funnel-shape structure. Scale: millimeter.

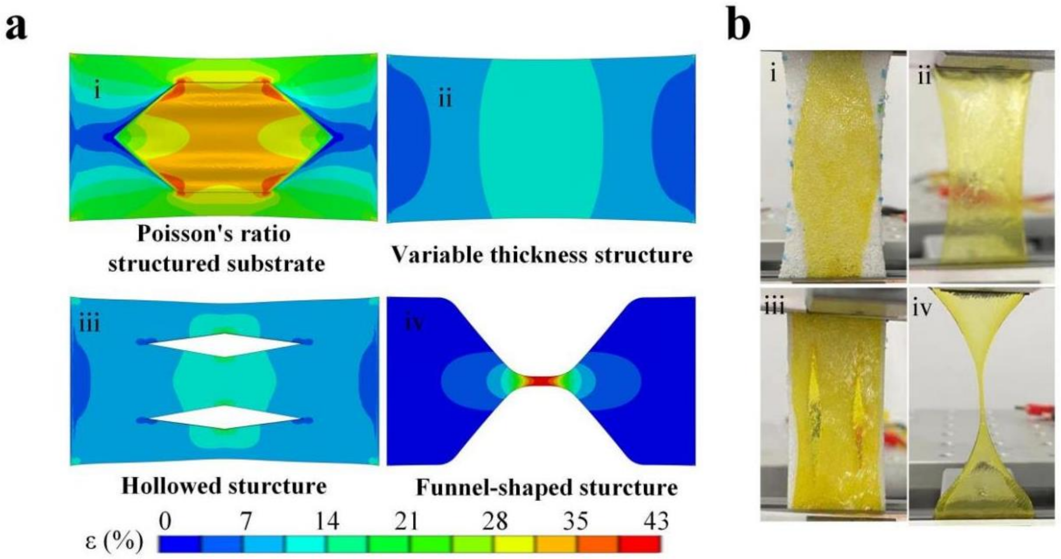

Figure S2. a) Strain distribution and b) diagram of heterogeneous structure designs for strain concentration.

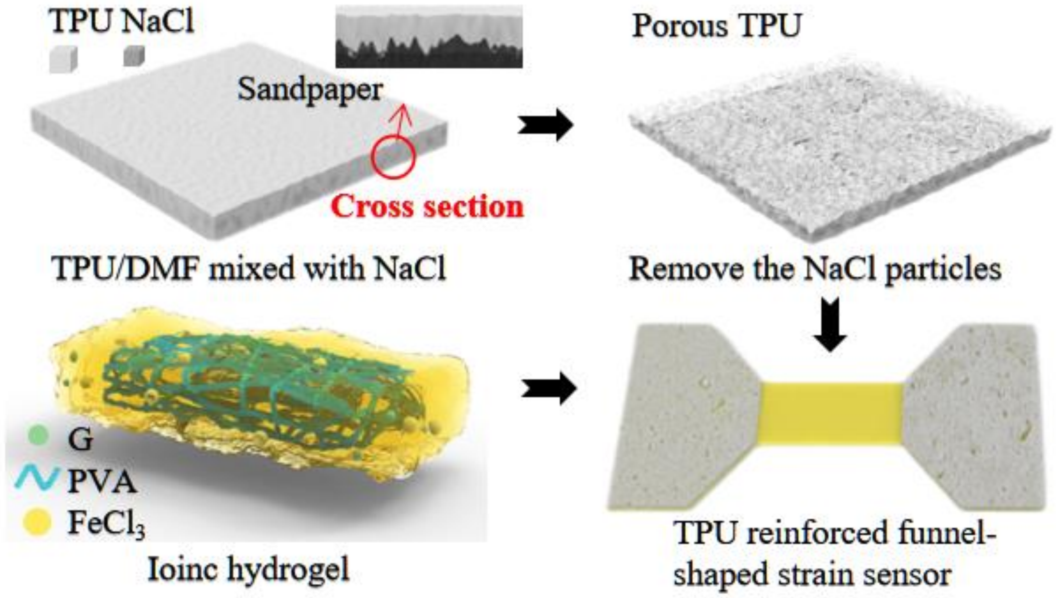

Figure S3. Schematic of the fabrication process of porous TPU and TPU-reinforced funnel-shaped IHSS.

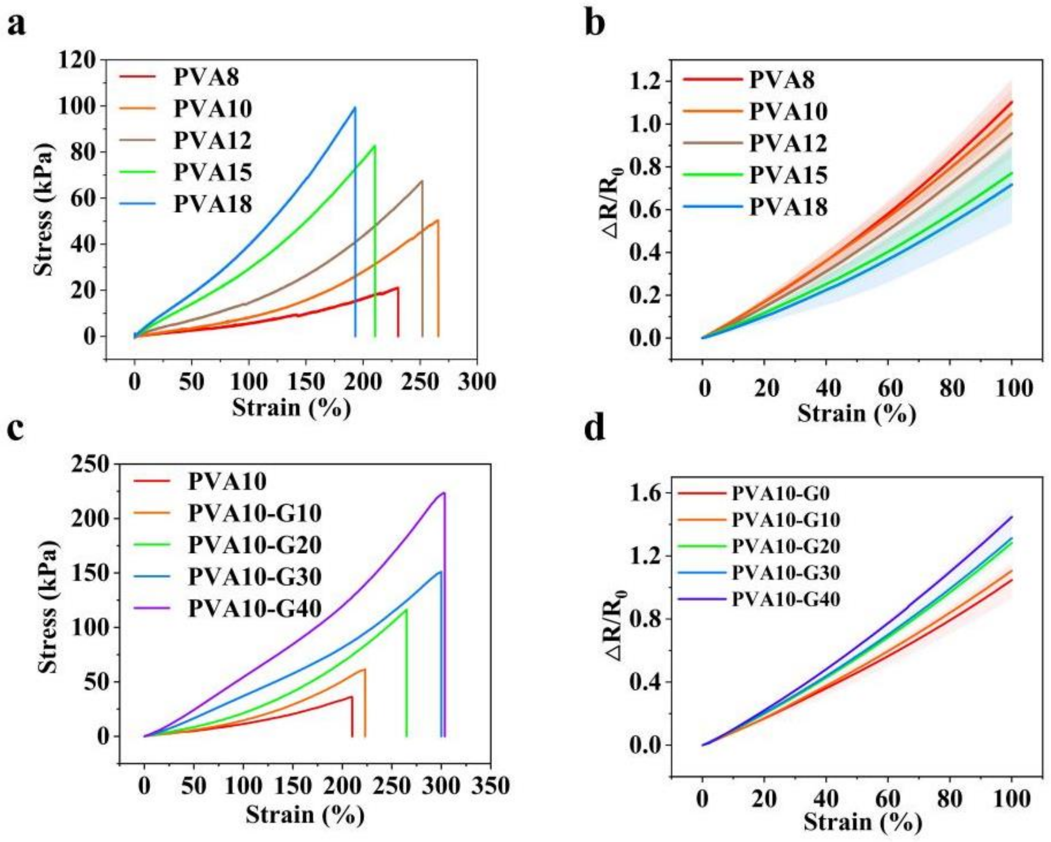

Figure S4. a) The stress-strain curve of PVAn hydrogels. b) The relative resistance change of PVAn hydrogels with the mass percentage of PVA varies from 8 wt% to 18 wt%. The strain range is 0-100 %. c) The stress-strain curve of PVA10-Gn hydrogels. d) The relative resistance change of PVA10-Gn hydrogels with mass percentage of G varies from 0 wt% to 40 wt%.

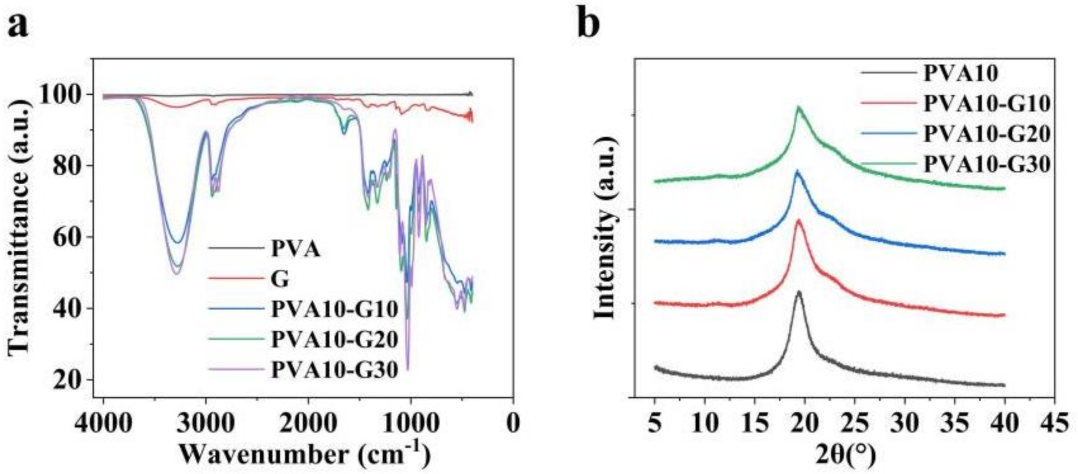

Figure S5. a) FT-IR curves of PVA powder, glycerol, and PVA10-Gn hydrogels. b) Typical XRD patterns of PVA hydrogel and PVA-Gn hydrogels in the dry state.

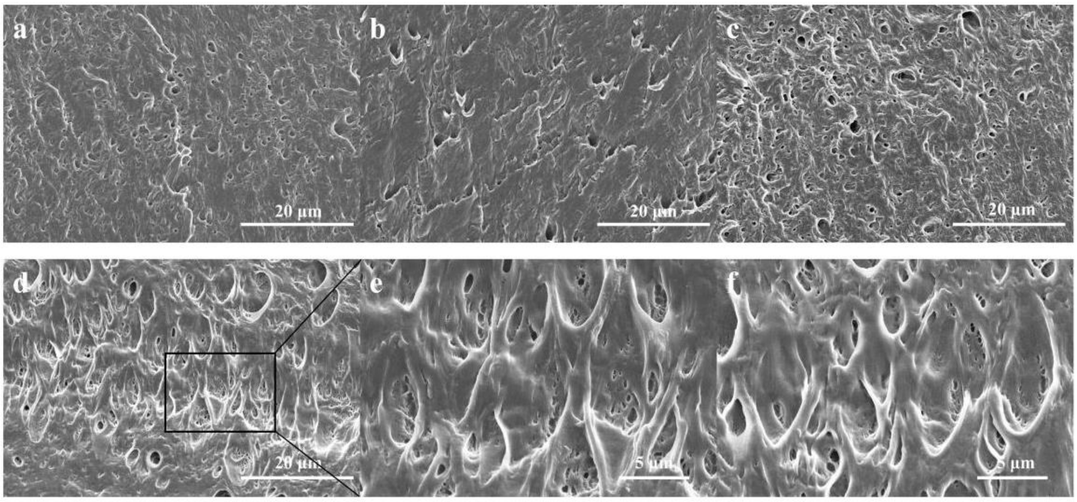

Figure S6. SEM images of a) PVA10, b) PVA10-G10, c) PVA10-G20, d) PVA10-G30, e) and f) PVA10-G30 at smaller scales.

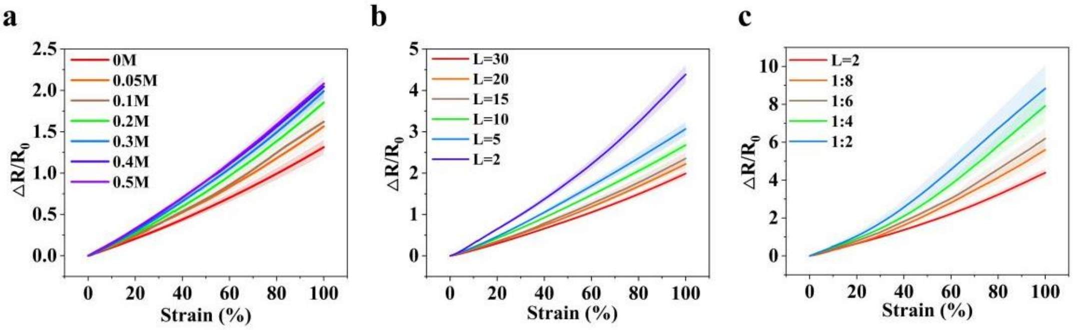

Figure S7. The relative resistance change of a) PVA10-G30-$FeCl_3$ hydrogels with a molar concentration of $FeCl_3$ varies from 0 M to 0.5 M, b) funnel-shaped PVA10-G30-$FeCl_3$ (0.3 M) hydrogels with L varies from 30 mm to 2 mm, and c) the TPU reinforced PVA10-G30-$FeCl_3$ hydrogels.

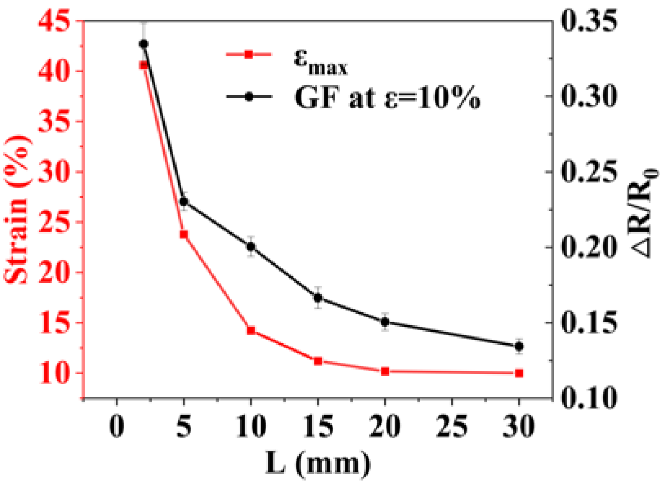

Figure S8. The maximum local strain (obtained by FEM) and GF of funnel-shaped ionic hydrogels under 10 % total strain.

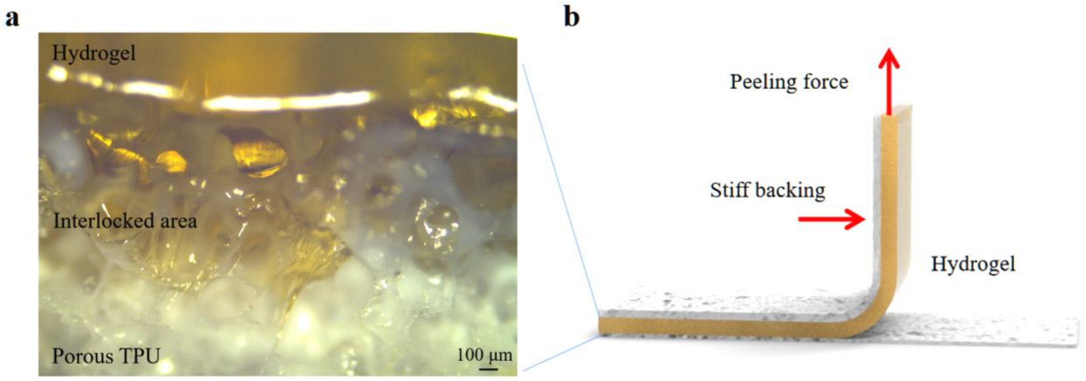

Figure S9. a) Hydrogel solution can soak into the micropores on the surface of the TPU layer, resulting in a large interfacial area. After frozen cross-linking, the hydrogel was mechanically interlocked at the surface of the porous TPU layer. b) The schematic diagram of the 90°-peeling test. A stiff backing is introduced to prevent elongation of the hydrogel sheet along the peeling direction.

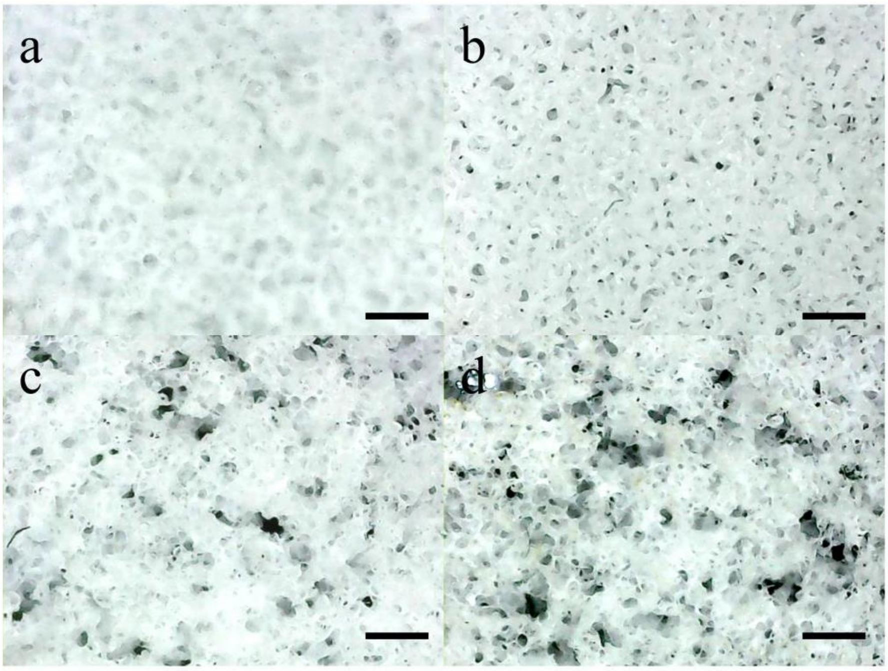

Figure S10. The optical microscope images of porous TPU with the TPU: NaCl ratio of a) 1:2, b) 1:4, c) 1:6 and d) 1:8. Scale bar: 1 mm.

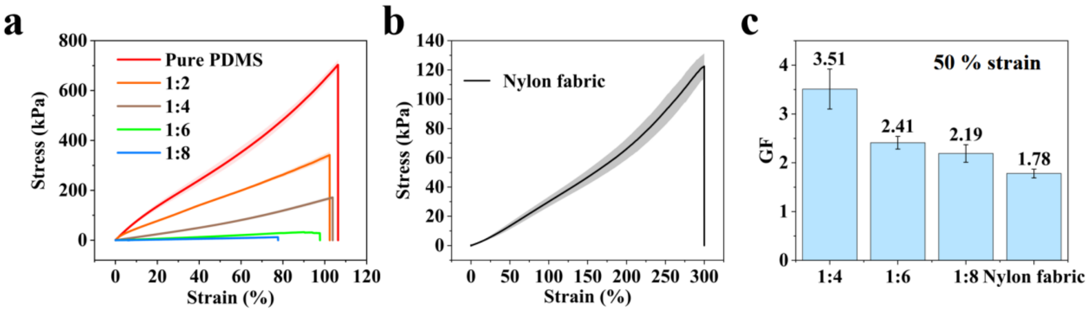

Figure S11. The stress-strain curve of a) pure PDMS and porous PDMS with different NaCl ratio and b) Nylon fabric. c) The GF of porous PDMS and Nylon fabric-reinforced funnel-shaped PVA10-G30-$FeCl_3$ hydrogel at 50 % strain. 100 % strain cannot be reached due to poor stretchability and weak interfacial bonding of hydrogel- PDMS hybrid.

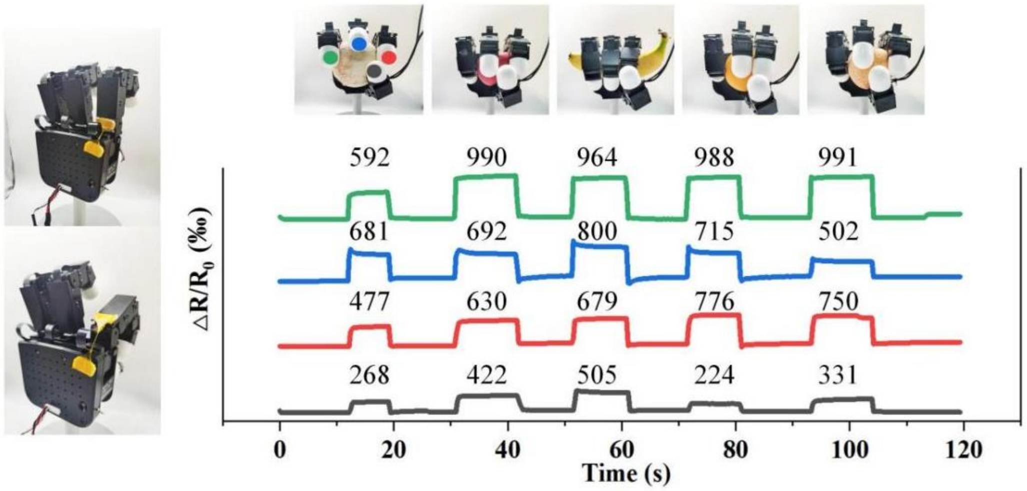

Figure S12. Fruit recognition with four-finger hand (Allegro) grasping by attaching the sensor to the joints.

Table S1. Comparisons of IHSS.

Ref.	Materials	Strain limit (%)	Gauge factor
This work	$FeCl_3$/glycerol/PVA[a]	310	1.31-9.21
Ref 3	Ionic liquid [BMIM][PF6][b]/LCE[c]	121	1.5
Ref 4a	NaCl/glycerol/PVA	355	1.56
Ref 4b	NaCl/glycerol/PVA	570	4
Ref 5b	$AlCl_3$/TA@HAP NWs[d]/ethylene glycol/PVA	350	2.84
Ref 13b	NaCl/HPC[e]/PVA	975	0.98
Ref 16a	Ionic liquid [DEIM][TFSI][f]/PU[g]	300	1.54
Ref 16b	Ionic liquid [EMIM][TFSI][h]/(P(VDF-co-HFP))[i]/(P(MMA-co-BMA))[j]	307	1.62
Ref 16c	Ionic liquid [BMIM][TFSI]/AAm[k]/AAc[l]/NIPAM[m]	200	0.8
Ref 16d	Ionic liquid [EMIM][TFSI]/PMMA[n]/PBA[o]	850	2.7
Ref 16e	chitosan/PAA[p]/TA@CNC[q]	800	3
Ref 16f	NaCl/PAM[r]	1200	4.58
Ref 16g	NaCl/sodium alginate/PAM	1800	3
Ref 16h	NaCl/PVA/gelatin/glycerin	650	0.8
Ref 16i	sodium alginate/TA/PAM	2100	2
Ref 16j	$FeCl_3$/PA[s]	700	2.43
Ref 16k	$(NH_4)_2S_2O_8$/allyl cellulose	126	0.3
Ref 16l	HCl/SBMA[t]/HEMA(P(SBMA-co-HEMA))[u]/PVA	300	3.36
Ref 16m	NaCl/Alg[v]/PAAm	1840	0.46
Ref 17a	NaCl/PAMAA[w]/glycerol/PVA	1000	8.3
Ref 17b	TA@CNC/PAA	2000	7.8
Ref 17c	KCl/ethylene glycol/glycerol/PAM/carrageenan	400	6

[a)] poly(vinyl alcohol), [b)] 1-butyl-3-methylimidazolium hexafluorophsphate, [c)] liquid crystal elastomer, [d)] tannic acid-coated hydroxyapatite nanowires, [e)] hydroxypropyl cellulose, [f)] 1,2-dimethyl-3-ethoxyethyl-imidazolium bis(trifluoromethanesulfonyl)imide, [g)]

poly(urea-urethane), h) 1-ethyl-3-methylimidazolium bis(trifluoromethylsulfonyl) imide, i) poly(vinylidene fluoride-co-hexafluoropropylene), j) poly(methyl methacrylate-co-butylmethacrylate), k) acrylamide, l) acrylic acid, m) n-isopropylacrylamide, n) poly(methyl methacrylate), o) poly(butyl acrylate), p) poly(acrylic acid), q) tannic acid-coated cellulose nanocrystals, r) polyampholyte, s) polyacrylamide, t) [2-(methacryloyloxy) ethyl]dimethyl-(3-sulfopropyl) ammonium hydroxide, u) 2-hydroxyethyl methacrylate, v) ionically cross-linked alginate, w) poly(acrylic amide-acrylic acid).

References

[1] B. D. Paulsen, K. Tybrandt, E. Stavrinidou, J. Rivnay, *Nat. Mater.* **2020**, 19, 13.
[2] M. Barrande, R. Bouchet, R. Denoyel, *Anal. Chem.* **2007**, 79, 9115.
[3] J.-H. Cao, B.-K. Zhu, Y.-Y. Xu, *J. Membrane Sci.* **2006**, 281, 446.
[4] Y. Shi, B. Li, Y. Zhang, Y. Cui, Z. Cao, Z. Du, J. Gu, K. Shen, S. Yang, *Adv. Energy Mater.* **2021**, 11, 2003663.
[5] M. Yao, B. Wu, X. Feng, S. Sun, P. Wu, *Adv. Mater.* **2021**, 33, 2103755.
[6] A. J. Bard, G. Inzelt, F. Scholz, *Electrochemical dictionary*, Springer, **2012**.
[7] C. Keplinger, J.-Y. Sun, C. C. Foo, P. Rothemund, G. M. Whitesides, Z. Suo, *Science* **2013**, 341, 984.
[8] L. Han, K. Liu, M. Wang, K. Wang, L. Fang, H. Chen, J. Zhou, X. Lu, *Adv. Funct. Mater.* **2018**, 28, 1704195.

Movie S1. Strain distribution of ionic hydrogels with homogeneous and heterogeneous structures.
Movie S2. Controlling the robot arm and claw to grasp objects through an orthogonal arranged controller.
Movie S3. A 3 × 3 IHSS array for sensing strain distribution.
